# COVID-19 Outbreak — New York City, February 29–June 1, 2020

**DOI:** 10.15585/mmwr.mm6946a2

**Published:** 2020-11-20

**Authors:** Corinne N. Thompson, Jennifer Baumgartner, Carolina Pichardo, Brian Toro, Lan Li, Robert Arciuolo, Pui Ying Chan, Judy Chen, Gretchen Culp, Alexander Davidson, Katelynn Devinney, Alan Dorsinville, Meredith Eddy, Michele English, Ana Maria Fireteanu, Laura Graf, Anita Geevarughese, Sharon K. Greene, Kevin Guerra, Mary Huynh, Christina Hwang, Maryam Iqbal, Jillian Jessup, Jillian Knorr, Julia Latash, Ellen Lee, Kristen Lee, Wenhui Li, Robert Mathes, Emily McGibbon, Natasha McIntosh, Matthew Montesano, Miranda S. Moore, Kenya Murray, Stephanie Ngai, Marc Paladini, Rachel Paneth-Pollak, Hilary Parton, Eric Peterson, Renee Pouchet, Jyotsna Ramachandran, Kathleen Reilly, Jennifer Sanderson Slutsker, Gretchen Van Wye, Amanda Wahnich, Ann Winters, Marcelle Layton, Lucretia Jones, Vasudha Reddy, Anne Fine

**Affiliations:** 1 New York City Department of Health and Mental Hygiene, Long Island City, New York.

New York City (NYC) was an epicenter of the coronavirus disease 2019 (COVID-19) outbreak in the United States during spring 2020 (*1*). During March–May 2020, approximately 203,000 laboratory-confirmed COVID-19 cases were reported to the NYC Department of Health and Mental Hygiene (DOHMH). To obtain more complete data, DOHMH used supplementary information sources and relied on direct data importation and matching of patient identifiers for data on hospitalization status, the occurrence of death, race/ethnicity, and presence of underlying medical conditions. The highest rates of cases, hospitalizations, and deaths were concentrated in communities of color, high-poverty areas, and among persons aged ≥75 years or with underlying conditions. The crude fatality rate was 9.2% overall and 32.1% among hospitalized patients. Using these data to prevent additional infections among NYC residents during subsequent waves of the pandemic, particularly among those at highest risk for hospitalization and death, is critical. Mitigating COVID-19 transmission among vulnerable groups at high risk for hospitalization and death is an urgent priority. Similar to NYC, other jurisdictions might find the use of supplementary information sources valuable in their efforts to prevent COVID-19 infections.

This report describes cases of laboratory-confirmed COVID-19 among NYC residents diagnosed during February 29–June 1, 2020, that were reported to DOHMH. DOHMH began COVID-19 surveillance in January 2020 when testing capacity for SARS-CoV-2 (the virus that causes COVID-19) using real-time reverse transcription–polymerase chain reaction (RT-PCR) was limited by strict testing criteria because of limited test availability only through CDC. The NYC and New York State public health laboratories began testing hospitalized patients at the end of February and early March. DOHMH encouraged patients with mild symptoms to remain at home rather than seek health care because of shortages of personal protective equipment and laboratory tests at hospitals and clinics. Commercial laboratories began testing for SARS-CoV-2 in mid- to late March. During February 29–March 15, patients with laboratory-confirmed COVID-19 were interviewed by DOHMH, and close contacts were identified for monitoring. The rapid rise in laboratory-confirmed cases (cases) quickly made interviewing all patients, as well as contact tracing, unsustainable. Subsequent case investigations first included medical chart review for patients who were hospitalized or who had died, but then progressed to chart review only for patients who had died, and then finally only for deaths in patients aged <65 years. On April 14, DOHMH began to report probable COVID-19–associated deaths (i.e., no known positive SARS-CoV-2 test result and death certificate listing cause of death as COVID-19 or an equivalent term [e.g., COVID, SARS-CoV-2, or another term]).

DOHMH quickly recognized the need for supplementary information sources and relied on direct data importation and matching of patient identifiers for data on hospitalization status, the occurrence of death, race/ethnicity, and presence of underlying medical conditions, including diabetes, lung disease, cancer, immunodeficiency, heart disease, asthma, kidney disease, gastrointestinal/liver disease, and obesity. These supplementary data systems included emergency department syndromic surveillance, the New York State Hospital Emergency Response Data System, regional health information organizations, NYC public hospitals, DOHMH’s electronic death registry system, and remote access to hospitals’ electronic health record systems. Even with these supplementary data sources, many variables (e.g., race/ethnicity) were still incomplete, given variable data quality.

Descriptive statistics were calculated using SAS software (version 9.4; SAS Institute). Age-adjusted rates were calculated using direct standardization for age and weighting by the U.S. 2000 standard population (*2*). Crude rates of cumulative cases, deaths, and testing per 100,000 population were mapped by modified U.S. Census Bureau ZIP code tabulation area* using ArcGIS software (version 10.6.1; ESRI). Neighborhood-level poverty was defined as the percentage of residents within a ZIP code with household incomes <100% of the federal poverty level, per the American Community Survey 2013–2017 (low: <10%, medium: 10%–19.9%, high: 20%–29.9%, very high: ≥30%). Population estimates (for 2018) for age, sex, borough (county) of residence, racial/ethnic group, and neighborhood poverty were produced by DOHMH using U.S. Census Bureau Population Estimate Program files (unpublished data, NYC DOHMH, 2020).^†^

During February 29–June 1, 2020, a total of 203,792 COVID-19 cases were diagnosed and reported^§^ among residents of NYC, including 54,211 (26.6%) in persons known to have been hospitalized and 18,679 (9.2%) in persons who died. The age-adjusted cumulative citywide incidences were 2,263 cases, 582 hospitalizations, and 198 deaths per 100,000 population. Case counts increased rapidly from a weekly mean of 274 diagnosed cases per day during the week of March 8 to a peak weekly mean of 5,132 cases per day by the week of March 29 (Figure 1). Hospital admissions also peaked the week of March 29 (weekly mean = 1,566 admissions per day). Deaths peaked during the week of April 5 (weekly mean = 566 per day). The median duration of hospitalization was 6 days (interquartile range [IQR] = 3–11 days). Among decedents with laboratory-confirmed COVID-19, the median interval from diagnosis to death was 8 days (IQR = 4–16 days). Among hospitalized patients, 32.1% were known to have died. The weekly proportion of hospitalized patients who died was highest among those admitted during March 22–April 5 (mean = 36.4%; range = 33.5%–38.2%).

**FIGURE 1:**
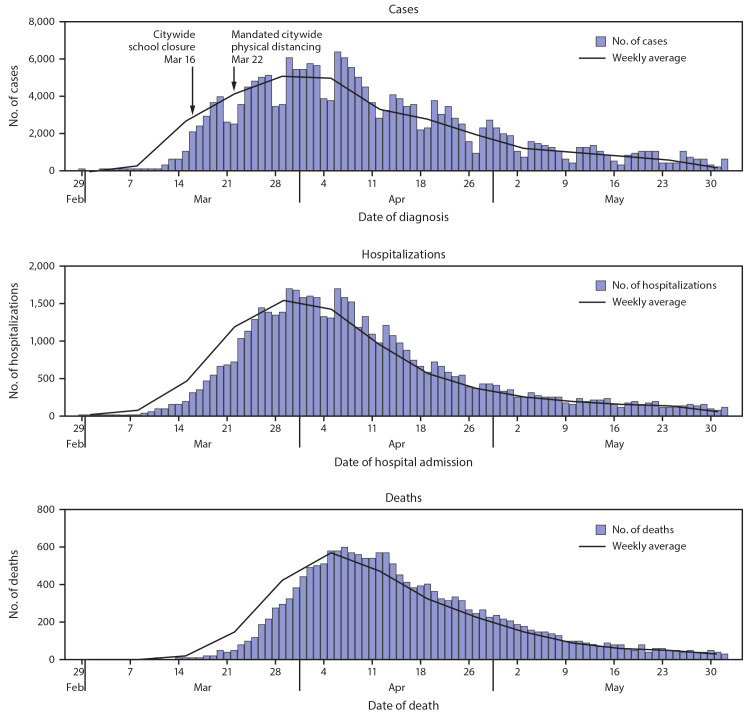
Daily laboratory-confirmed COVID-19 cases, associated hospitalizations, and deaths — New York City, February 29–June 1, 2020 **Abbreviation:** COVID-19 = coronavirus disease 2019.

Age-specific incidence was highest among adults aged 45–64 years (7,007 per 100,000) (Table). Hospitalization and death rates were highest among patients aged ≥75 years (2,146 and 1,311 per 100,000, respectively); among persons aged ≥75 years with confirmed cases, 38.3% were known to have died. Age-adjusted incidence, hospitalization rate, and death rate were higher among males than females, and all increased with increasing levels of neighborhood poverty. By borough, age-adjusted incidence, hospitalization rate, and death rate were consistently highest in the Bronx and lowest in Manhattan. Among the race/ethnicity groups with known identity, incidence was highest among Black/African American (Black) persons (1,590 per 100,000). Age-adjusted rates of hospitalization and death were highest among Black (699 and 248 per 100,000, respectively) and Hispanic/Latino (Hispanic) persons (658 and 260 per 100,000, respectively).

**TABLE:** Characteristics of cumulative laboratory-confirmed COVID-19 cases, hospitalizations, and deaths among New York City residents reported to the New York City Department of Health and Mental Hygiene — New York City, February 29–June 1, 2020*

Characteristic	Cases		Hospitalizations		Deaths	
	No.	Rate^†^	No. (row %)	Rate^†^	No. (row %)	Rate^†^
**Total**	**203,792**	**2,263**	**54,211 (26.6)**	**582**	**18,679 (9.2)**	**198**
**Age group, yrs**						
0–17	6,016	348	508 (8.4)	29	12 (0.2)	1
18–44	74,654	2,215	8,474 (11.4)	251	686 (0.9)	20
45–64	73,998	7,007	18,219 (24.6)	1,725	4,183 (5.7)	396
65–74	25,182	2,518	12,009 (47.7)	1,201	4,634 (18.4)	463
≥75	23,942	3,425	15,001 (62.7)	2,146	9,164 (38.3)	1,311
**Sex**						
Female	98,992	2,060	23,612 (23.9)	456	7,494 (7.6)	136
Male	104,675	2,511	30,589 (29.2)	744	11,183 (10.7)	283
**Race/Ethnicity**						
Hispanic/Latino	36,498	1,514	15,288 (41.9)	658	5,743 (15.7)	260
Black/African American	32,458	1,590	14,676 (45.2)	699	5,215 (16.1)	248
White	31,029	988	11,057 (35.6)	314	4,745 (15.3)	123
Asian/Pacific Islander	8,122	601	3,441 (42.4)	258	1,403 (17.3)	111
American Indian/Alaska Native	196	973	33 (16.8)	168	5 (2.6)	27
Other race/Missing	95,489	—^§^	9,716 (10.2)	—^§^	1,568 (1.6)	—^§^
**Neighborhood poverty^¶^**						
Low	33,114	1,787	7,498 (22.6)	358	2,756 (8.3)	125
Medium	79,327	2,169	20,907 (26.4)	551	7,404 (9.3)	193
High	48,998	2,315	15,034 (30.7)	700	5,184 (10.6)	241
Very high	36,642	2,706	10,341 (28.2)	796	3,305 (9)	268
**Borough of residence**						
Bronx	46,085	3,157	12,076 (26.2)	826	3,870 (8.4)	268
Brooklyn	56,548	2,104	15,125 (26.7)	556	5,563 (9.8)	205
Manhattan	25,315	1,369	7,867 (31.1)	408	2,476 (9.8)	123
Queens	62,260	2,507	16,806 (27)	637	5,882 (9.4)	217
Staten Island	13,577	2,701	2,337 (17.2)	423	888 (6.5)	158

**Abbreviation:** COVID-19 = coronavirus disease 2019.

* Data missing on sex for 138 persons, on borough for nine persons, and on neighborhood poverty for 6,660 persons.

^†^ Per 100,000 population; rates for sex, race/ethnicity, neighborhood poverty, and borough of residence were age-adjusted.

^§^ Rates not calculated because no population denominator.

^¶^ Neighborhood-level poverty was defined as the percentage of residents in a ZIP code with household incomes <100% of the federal poverty level, per the American Community Survey 2013–2017. Low poverty: <10%; medium poverty: 10%–19.9%; high poverty: 20%–29.9%; very high poverty: ≥30%.

Some neighborhoods with high case rates also had high testing rates (e.g., North Bronx and Northwest Queens) (Figure 2). However, other neighborhoods had low or medium testing rates and high percent positivity with medium to high case rates (Southeast Queens, East Brooklyn, West Bronx, and Northern Manhattan), suggesting possible underascertainment of cases. Citywide, the percentage of tests with positive results increased from 27% the week of March 8 to a peak of 65% during the week of March 22. The growth of testing rates lagged behind the growth of percent positivity but increased steadily from 86 per 100,000 during the week of March 8 to 1,634 per 100,000 by the week of May 24.

**FIGURE 2:**
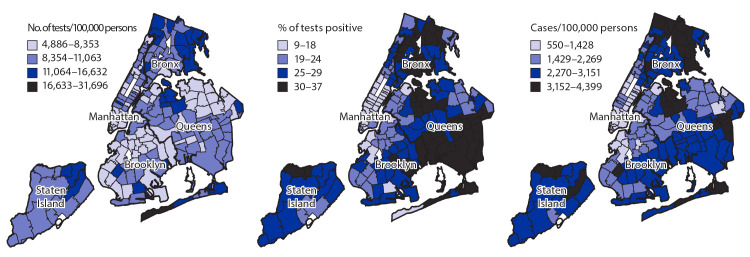
Cumulative crude rates of COVID-19 testing per 100,000 population, percentage of tests positive for SARS-CoV-2, and cumulative crude rates of COVID-19 cases per 100,000 population,* by modified ZIP code tabulation areas — New York City, February 29–June 1, 2020 **Abbreviation:** COVID-19 = coronavirus disease 2019. * All data are displayed by four levels of natural breaks.

Among 85% of decedents with known underlying medical conditions, the majority (75%) of decedents with a confirmed laboratory test had two or more underlying conditions; heart disease (73%), diabetes (58%), and chronic kidney disease (23%) were the most commonly reported conditions (NYC DOHMH, unpublished data; 2020). During March 11–June 1, 4,516 probable COVID-19–associated deaths were known to have occurred among NYC residents. These deaths occurred more commonly at home (30%) or in a nursing home (26%), compared with confirmed COVID-19 deaths (4% at home and 8% in a nursing home). Deaths occurring in a hospital were frequently laboratory-confirmed as COVID-19–associated (86%). Among 23,195 probable and confirmed deaths, 22.5% (5,226) were known to have occurred among residents of a nursing home.

## Discussion

Phylogenetic analysis and sentinel surveillance suggest that the introduction of COVID-19 into NYC from travelers started during early to mid-February 2020 (*3*,*4*), although the first case of laboratory-confirmed COVID-19 in NYC was diagnosed on February 29. The subsequent 3-month period was characterized by a rapid acceleration in the epidemic, resulting in approximately 203,000 cases and 18,600 deaths among persons with laboratory-confirmed COVID-19. Reported diagnoses of cases peaked 1 week after physical distancing orders were enacted (March 22). The overall crude case fatality rate of 9.2% is an overestimate because of underascertainment of cases, given the restrictive testing guidance and limited availability of tests for the first 2 months of the epidemic.^¶^ Similar to findings from the United Kingdom,** approximately 30% of hospitalized patients with laboratory-confirmed COVID-19 were known to have died. The increased case fatality rate among hospitalized patients during the peak period of reported cases suggests that health care system capacity constraints might have influenced patient outcomes.

As has been previously reported (*5*), COVID-19 incidence and related hospitalization and mortality were elevated among Black and Hispanic persons and among residents of high-poverty neighborhoods. The finding of neighborhoods with low testing rates and a high percentage of positive test results suggests barriers to accessing testing in areas with considerable community transmission.

The rapid spread of COVID-19, combined with a lack of testing availability early in 2020, led to considerable surveillance challenges. DOHMH quickly ceased labor-intensive individual case investigations for all patients and sought supplementary sources of information. In addition, publishing NYC DOHMH data online in real-time^††^ allowed the public to access basic and important information on COVID-19 in NYC.

The findings in this report are subject to at least four limitations. First, these data are based primarily on laboratory-confirmed disease, which is more likely to represent severe illness, especially early in the epidemic when COVID-19 testing was mostly limited to hospitalized patients. Second, hospitalizations were underestimated because of incomplete ascertainment from external sources. Third, race and ethnicity information was missing for a large proportion of nonhospitalized, nonfatal cases. Finally, rates are likely underestimated for more affluent neighborhoods because denominators do not reflect the differential exodus of wealthy NYC residents (*6*).

The initial wave of COVID-19 in NYC demonstrated that persons who were older, had underlying medical conditions, or resided in poorer neighborhoods, and racial and ethnic minority populations suffered disproportionately from SARS-Cov-2 infection and death. These trends represent the downstream effect of long-term policies, practices, attitudes, and cultural messages that promote, reinforce, and fail to eliminate inequities (*7*). In addition, Black and Hispanic persons are disproportionately employed in lower-paid, often frontline industries and occupations, work with limited ability to social distance, and are more likely to lack employer-based health insurance (*8*). Mitigating future morbidity and mortality from COVID-19 across NYC in the absence of a vaccine,^§§^ particularly among persons who are at increased risk, is an urgent priority.

**Summary**

**What is already known about this topic?**

New York City (NYC) was an early epicenter of the COVID-19 pandemic in the United States.

**What is added by this report?**

Approximately 203,000 cases of laboratory-confirmed COVID-19 were reported in NYC during the first 3 months of the pandemic. The crude fatality rate among confirmed cases was 9.2% overall and 32.1% among hospitalized patients. Incidence, hospitalization rates, and mortality were highest among Black/African American and Hispanic/Latino persons, as well as those who were living in neighborhoods with high poverty, aged ≥75 years, and with underlying medical conditions.

**What are the implications for public health practice?**

Mitigating COVID-19 transmission among vulnerable groups at high risk for hospitalization and death is an urgent priority.

## Footnotes

* https://www.census.gov/programs-surveys/geography/guidance/geo-areas/zctas.html.

† Population estimates were modified from U.S. Census Bureau interpolated intercensal population estimates, 2000–2018, updated August 2019.

§ As of July 27, 2020.

¶ https://www.medrxiv.org/content/10.1101/2020.06.27.20141689v1.full.pdf.

** https://www.medrxiv.org/content/10.1101/2020.04.23.20076042v1.full.pdf.

†† https://www1.nyc.gov/site/doh/covid/covid-19-data.page.

§§ https://www.cdc.gov/coronavirus/2019-ncov/community/health-equity/index.html.
