# *Erratum:* Vol. 69, No. 46

**DOI:** 10.15585/mmwr.mm6950a7

**Published:** 2020-12-18

In the report “COVID-19 Outbreak — New York City, February 29–June 1, 2020,” on p. 1725, the list of authors should have read “Corinne N. Thompson, PhD^1^; Jennifer Baumgartner, MSPH^1^; Carolina Pichardo^1^; Brian Toro^1^; Lan Li, MPH^1^; Robert Arciuolo, MPH^1^; Pui Ying Chan, MPH^1^; Judy Chen^1^; Gretchen Culp, PhD^1^; Alexander Davidson, MPH^1^; Katelynn Devinney, MPH^1^; Alan Dorsinville, MPH^1^; Meredith Eddy, MPH^1^; Michele English^1^; Ana Maria Fireteanu, MPH^1^; Laura Graf, MPH^1^; Anita Geevarughese, MD^1^; Sharon K. Greene, PhD^1^; Kevin Guerra, MPH^1^; Mary Huynh, PhD^1^; Christina Hwang, MPH^1^; Maryam Iqbal, MPH^1^; Jillian Jessup, MPH^1^; Jillian Knorr, MPH^1^; **Ramona Lall, PhD^1^;** Julia Latash, MPH^1^; Ellen Lee, MD^1^; Kristen Lee, MPH^1^; Wenhui Li, PhD^1^; Robert Mathes, MPH^1^; Emily McGibbon, MPH^1^; Natasha McIntosh^1^; Matthew Montesano, MPH^1^; Miranda S. Moore, MPH^1^; Kenya Murray, MPH^1^; Stephanie Ngai, MPH^1^; Marc Paladini, MPH^1^; Rachel Paneth-Pollak, MD^1^; Hilary Parton, MPH^1^; Eric Peterson, MPH^1^; Renee Pouchet, MHA^1^; Jyotsna Ramachandran, MPH^1^; Kathleen Reilly, PhD^1^; Jennifer Sanderson Slutsker, MPH^1^; Gretchen Van Wye, PhD^1^; Amanda Wahnich, MPH^1^; Ann Winters, MD^1^; Marcelle Layton, MD^1^; Lucretia Jones, DrPH^1^; Vasudha Reddy, MPH^1^; Anne Fine, MD^1^.”

The affiliation for Dr. Lall is New York City Department of Health and Mental Hygiene, Long Island City, New York.

